# Predictors of human immunodeficiency virus and tuberculosis co-infection

**DOI:** 10.4178/epih/e2015007

**Published:** 2015-02-16

**Authors:** Venkataramana Kandi

**Affiliations:** Department of Microbiology, Prathima Institute of Medical Sciences, Karimnagar, India

**Dear Editor:**

The original paper by Molaeipoor et al. [1] has come at just the right time. While the availability of highly active antiretroviral therapy (HAART) has come as a boon for the human immunodeficiency virus (HIV)-infected population and is instrumental in prolonging life and improving its quality, the co-morbidities associated with HIV remain as a cause for concern. A previous study has noted that HIV-seropositive patients have significantly higher chances of developing other infections like tuberculosis (TB) [2]. The HIV disease course is influenced by the presence of co-morbidities that include infectious diseases like TB, hepatitis B, hepatitis C, and other infectious and non-infectious conditions including malignancies [3,4]. A recent study has also observed that the disease burden in HIV patients is significantly related to illicit drug use [5]. It must be noted that identification of various co-morbidities and their underlying causes prior to initiation of HAART is necessary to minimise related additional complications and resultant morbidity and mortality. The results of Molaeipoor et al. [1] indicating that overcrowding (e.g., in jails), adverse effects of HAART, prior latent TB infection, TCD4^+^ counts lower than 350 cells/mm^3^, and prophylactic therapy against other infections were more instrumental in predisposing HIV-seropositive patients to TB than was drug abuse are very significant findings. These results further suggest that future research should concentrate on nutritional issues (e.g., malnutrition) in the HIV-infected population, which may include vitamin and mineral deficiencies.
